# Guanosine modulates SUMO2/3-ylation in neurons and astrocytes via adenosine receptors

**DOI:** 10.1007/s11302-020-09723-0

**Published:** 2020-09-05

**Authors:** Camila A. Zanella, Carla I. Tasca, Jeremy M. Henley, Kevin A. Wilkinson, Helena I. Cimarosti

**Affiliations:** 1 Department of Pharmacology, Federal University of Santa Catarina (UFSC), Florianópolis, SC Brazil; 2 Department of Biochemistry, UFSC, Florianópolis, SC Brazil; 3 School of Biochemistry, University of Bristol, Bristol, UK

**Keywords:** Astrocytes, Neurons, Post-translational modification, Purinergic system, SUMO

## Abstract

SUMOylation is a post-translational modification (PTM) whereby members of the Small Ubiquitin-like MOdifier (SUMO) family of proteins are conjugated to lysine residues in target proteins. SUMOylation has been implicated in a wide range of physiological and pathological processes, and much attention has been given to its role in neurodegenerative conditions. Due to its reported role in neuroprotection, pharmacological modulation of SUMOylation represents an attractive potential therapeutic strategy in a number of different brain disorders. However, very few compounds that target the SUMOylation pathway have been identified. Guanosine is an endogenous nucleoside with important neuromodulatory and neuroprotective effects. Experimental evidence has shown that guanosine can modulate different intracellular pathways, including PTMs. In the present study we examined whether guanosine alters global protein SUMOylation. Primary cortical neurons and astrocytes were treated with guanosine at 1, 10, 100, 300, or 500 μM at four time points, 1, 6, 24, or 48 h. We show that guanosine increases global SUMO2/3-ylation in neurons and astrocytes at 1 h at concentrations above 10 μM. The molecular mechanisms involved in this effect were evaluated in neurons. The guanosine-induced increase in global SUMO2/3-ylation was still observed in the presence of dipyridamole, which prevents guanosine internalization, demonstrating an extracellular guanosine-induced effect. Furthermore, the A1 adenosine receptor antagonist DPCPX abolished the guanosine-induced increase in SUMO2/3-ylation. The A2A adenosine receptor antagonist ZM241385 increased SUMOylation per se, but did not alter guanosine-induced SUMOylation, suggesting that guanosine may modulate SUMO2/3-ylation through an A1-A2A receptor interaction. Taken together, this is the first report to show guanosine as a SUMO2/3-ylation enhancer in astrocytes and neurons.

**Electronic supplementary material:**

The online version of this article (10.1007/s11302-020-09723-0) contains supplementary material, which is available to authorized users.

## Introduction

SUMOylation is a post-translational modification (PTM) whereby the Small Ubiquitin-like MOdifier (SUMO) peptide is conjugated to target proteins at lysine residues [1]. SUMO conjugation to target proteins is mediated by a three-step, ATP-dependent enzymatic cascade involving E1, E2, and E3 enzymes, and can be reversed by the actions of SUMO proteases, the most well characterized of which are the SENP family [1]. SUMOylation plays important physiological roles [2, 3] and, in neurons, has been shown to be crucial for synaptic plasticity and cellular communication [4–7]. We and others have shown that SUMOylation is part of an endogenous neuroprotective response in ischemic conditions [8–12]. Furthermore, several proteins implicated in ischemia [13], and neurodegenerative disorders [14], such as Alzheimer’s [15–17] and Parkinson’s diseases [18, 19], are SUMO targets, and SUMOylation has been linked to age-related processes [20, 21]. As a result, SUMOylation may represent an attractive therapeutic target in several disorders. However, relatively few compounds that can target protein SUMOylation have so far been identified.

Accumulating evidence has demonstrated that guanosine, an endogenous nucleoside, may be a therapeutically useful compound in a number of disorders [22–24]. Due to its role in fundamental cellular mechanisms, guanosine promotes many protective effects such as anti-inflammatory effects during aging in astrocytes [25] and is protective against in vivo amyloid-beta (Aβ)-induced toxicity [26], seizures [27], and ischemia [28, 29]. However, despite several studies reporting the protective effects of guanosine [22–24], little is known about the molecular mechanisms involved. We have recently shown that guanosine can prevent ischemia-induced increases in reactive oxygen species (ROS) and impairment of glutamate uptake [29]. Guanosine-mediated phosphorylation and, consequently, activation of Akt/PKB and inactivation of glycogen synthase kinase 3β (GSK3β) seem to be crucial for its anti-apoptotic effects under cellular stress conditions caused by oxidative damage [30], glutamate [31], and staurosporine [32]. In addition, guanosine can stimulate neural stem cell proliferation via phosphorylation/activation of CREB [33], further suggesting that phosphorylation of proteins might contribute the molecular effects of guanosine.

Although a specific receptor for guanosine has not been identified [34], a number of reports suggest it may interact with adenosine receptors (A1 and A2A) [24, 29, 35–38]. Furthermore, the neuroprotective effect of guanosine was also suggested to be through the large conductance Ca^2+^-activated K^+^ channel (BK) [30].

Here, we sought to evaluate whether guanosine may modulate global protein SUMOylation in neurons and astrocytes, and further determine whether adenosine receptors mediate these effects.

## Methods

### Cell culture

Cortical neurons were prepared as described previously [39]. Briefly, cortices from E18 Wistar rats were dissected in Hank’s balanced salt solution (HBSS, Gibco) followed by trypsin and mechanical dissociation. Neurons (55 × 10^4^ cells/well) were plated on 6-well plates previously treated with poly-L-lysine (0.1 mg/mL, Sigma). Plating medium consisted of Neurobasal Medium (Gibco) containing 10% horse serum (Gibco), B27 (1×, Gibco), penicillin-streptomycin (P/S, 100 units penicillin and 0.1 mg/mL streptomycin; Thermo Scientific), and 5 mM Glutamax (Gibco). After 24 h, plating medium was replaced with 3 mL of feeding medium (Neurobasal Medium, B27, P/S, Glutamax) with no further medium changes. Neurons were used for experiments at 14 days in vitro*.* Cortical astrocytes were prepared as described previously [40]. Briefly, cortices from Wistar rats (0–2 days old) were dissected in PBS (1×, containing 1 mM glucose) followed by mechanical dissociation. Astrocytes (70 × 10^4^ cells per well) were plated on 6-well plates previously treated with poly-L-lysine (0.1 mg/mL, Sigma). Plating medium consisted of Dulbecco’s modified Eagle’s medium (DMEM) nutrient mixture F-12 (Gibco), supplemented with 10% fetal bovine serum (Gibco). Cell culture medium was changed 24 h after plating and changed subsequently three times a week. Astrocytes were used for experiments at 14 days in vitro. Ethics committees previously approved all procedures used in this study (CEUA 955 – UFSC and UB/18/004 – University of Bristol).

### Drug treatments

Unless otherwise specified, the drugs used in the experiments were obtained from Sigma: guanosine (G6752), adenosine (A9251), dipyridamole (10 μM, D9766), DPCPX (100 nM, C101) and ZM 241385 (50 nM, Z0153). The concentration curves (1, 10, 100, 300 and 500 μM) for guanosine, adenosine and guanine were based on previous studies [29, 30, 36, 41, 42].

### Western blotting

For immunoblotting, neurons and astrocytes were lysed in 250 μL sample buffer solution (1×) containing 2% SDS (w/v), 5% glycerol (v/v), 62.5 mM Tris-HCl pH 6.8, and 5% (v/v) β-mercaptoethanol. Lysates were collected and heated to 95 °C for 10 min prior to gel electrophoresis. Proteins were separated by SDS-PAGE (10–15% gels). PDVF membranes were blocked in 5% (w/v) non-fat milk powder or bovine serum albumin (BSA, Sigma) in PBS-T. The following primary antibodies were incubated overnight at 4 °C: SUMO1 (1:1000, Cell Signaling, 4930), SUMO2/3 (1:1000, Cell Signaling, 4971S), SENP3 (1:1000, Cell Signaling, D20A10), and GAPDH (1:10000, Abcam, ab8248). Ponceau S (0.1% in 5% acetic acid, Sigma, P7170) was also used for protein staining [43]. After three washes with PBS-T, membranes were incubated with the following HRP-conjugated secondary antibodies for 1 h at room temperature: anti-mouse (1:5000, Abcam, ab6728) or anti-rabbit (1:5000, Cell Signaling, 7074S). After three washes of 5 min each in PBS-T, proteins were visualized by enhanced chemiluminescence (Thermo Scientific). Protein bands were quantified by densitometry using ImageJ software (NIH) [44].

### Immunofluorescence

For immunofluorescence, neurons and astrocytes were plated on glass coverslips (1 × 10^5^ cells/well) previously treated with nitric acid and poly-L-lysine (0.1 mg/mL). Neurons were washed with PBS once and fixed with 4% paraformaldehyde (PFA) for 20 min. After three washes with PBS, PFA was quenched by incubation with PBS containing 20 mM glycine for 10 min, before cells were permeabilized with PBS containing 0.05% Triton for 5 min. Next, blocking solution consisting of 5% BSA diluted in PBS was added to the coverslips for 1 h at room temperature. Neurons were then incubated with anti-β-tubulin III (1:250, Sigma, T2200) and astrocytes with anti-GFAP (1:100; Sigma-Aldrich, 3670S) for 1 h. Cells were then washed twice with PBS containing 0.1% tween 20 for 5 min and incubated with Alexa Fluor 594 fluorescent antibodies (1:100, Invitrogen, A32740) for 1 h at room temperature. For nuclear staining, Hoechst 33342 was present in the mounting media. Images were acquired on a confocal microscope (Leica DMI6000 B, LCME-UFSC). To analyze morphology, three independent experiments were performed [29, 45].

### MTT assay

Cell viability was assessed by the colorimetric MTT (3-(4,5-dimethylthiazol-2-yl)-2,5-diphenyltetrazolium bromide) assay [46]. After the respective treatments, neurons and astrocytes were incubated with MTT (0.2 mg/mL) diluted in PBS and kept at 37 °C for 2 h. MTT was removed and DMSO (100%) added to cells. The absorbance was read at 540 nm in 96-well plates.

### Statistical analysis

All results were included in the statistical analysis except for those significantly detected as outliers (https://www.graphpad.com/quickcalcs/Grubbs1.cfm). After confirming data normality (Kolmogorov-Smirnov test), one-way analysis of variance (ANOVA) was performed and Newman-Keuls was used as a post hoc test to determine significant differences among groups. Data are presented as mean + standard error of the mean (S.E.M.) and statistical significance expressed by *p ≤ 0.05, **p ≤ 0.01, and ***p ≤ 0.001. GraphPad Prism 5.0 was used for graphs and statistics [44, 47].

## Results

### Guanosine increases SUMO2/3 conjugation in neurons and astrocytes

**Fig. 1:**
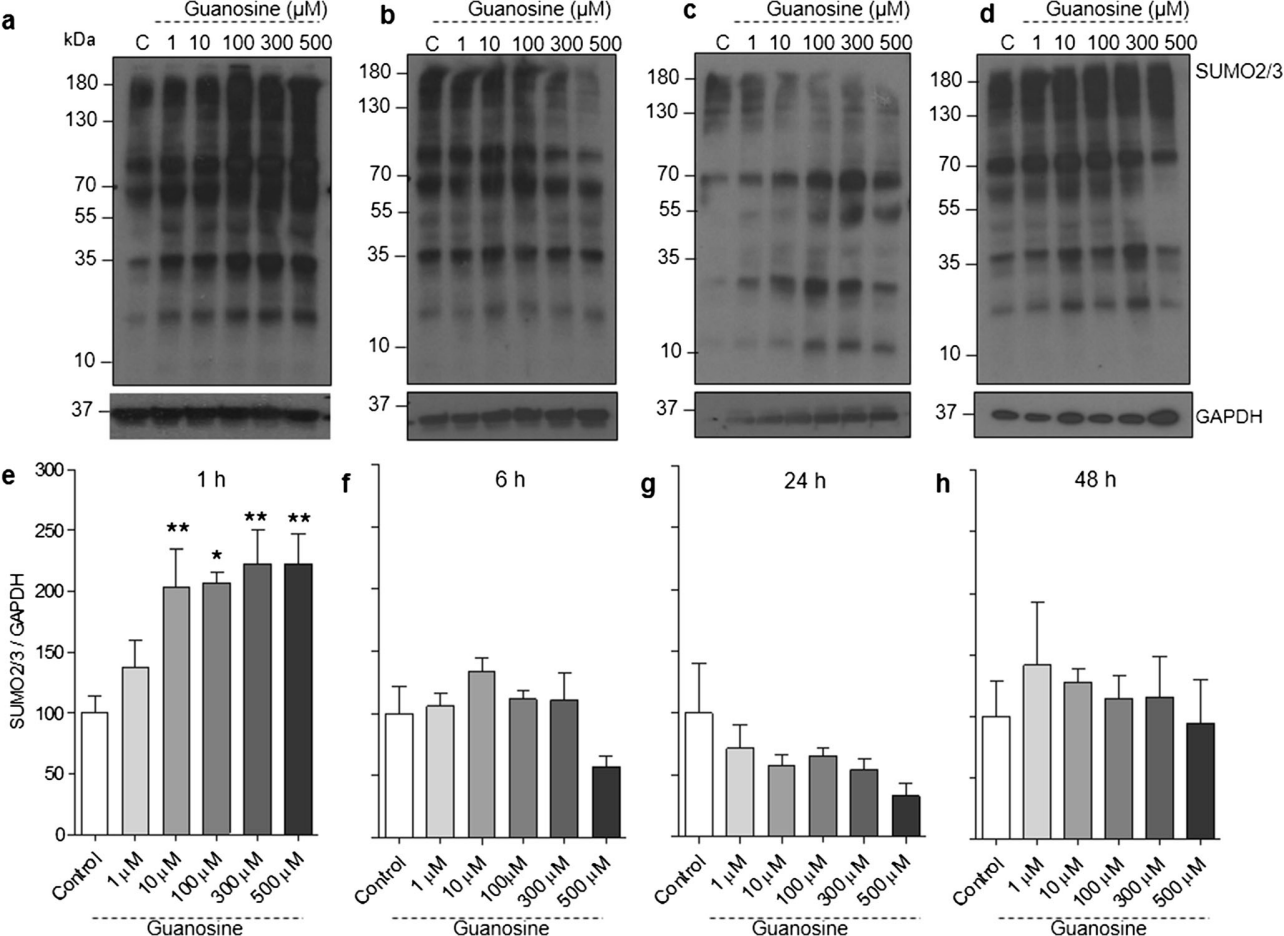
Guanosine increases global SUMO2/3 conjugation in neurons. Representative SUMO2/3 Western blots from neurons treated with guanosine (1–500 μM) for **a** 1 h, **b** 6 h, **c** 24 h, and **d** 48 h. **b** Optical density quantification of global SUMO2/3 conjugation for **e** 1 h, **f** 6 h, **g** 24 h, and **h** 48 h. GAPDH was used as a loading control. Results expressed as mean + standard error of the mean (*n* = 3–5 independent experiments). One-way ANOVA followed by Newman-Keuls multiple comparison analyses (*p < 0.05, **p < 0.01 vs. control). C, control; kDa, kilodaltons

**Fig. 2:**
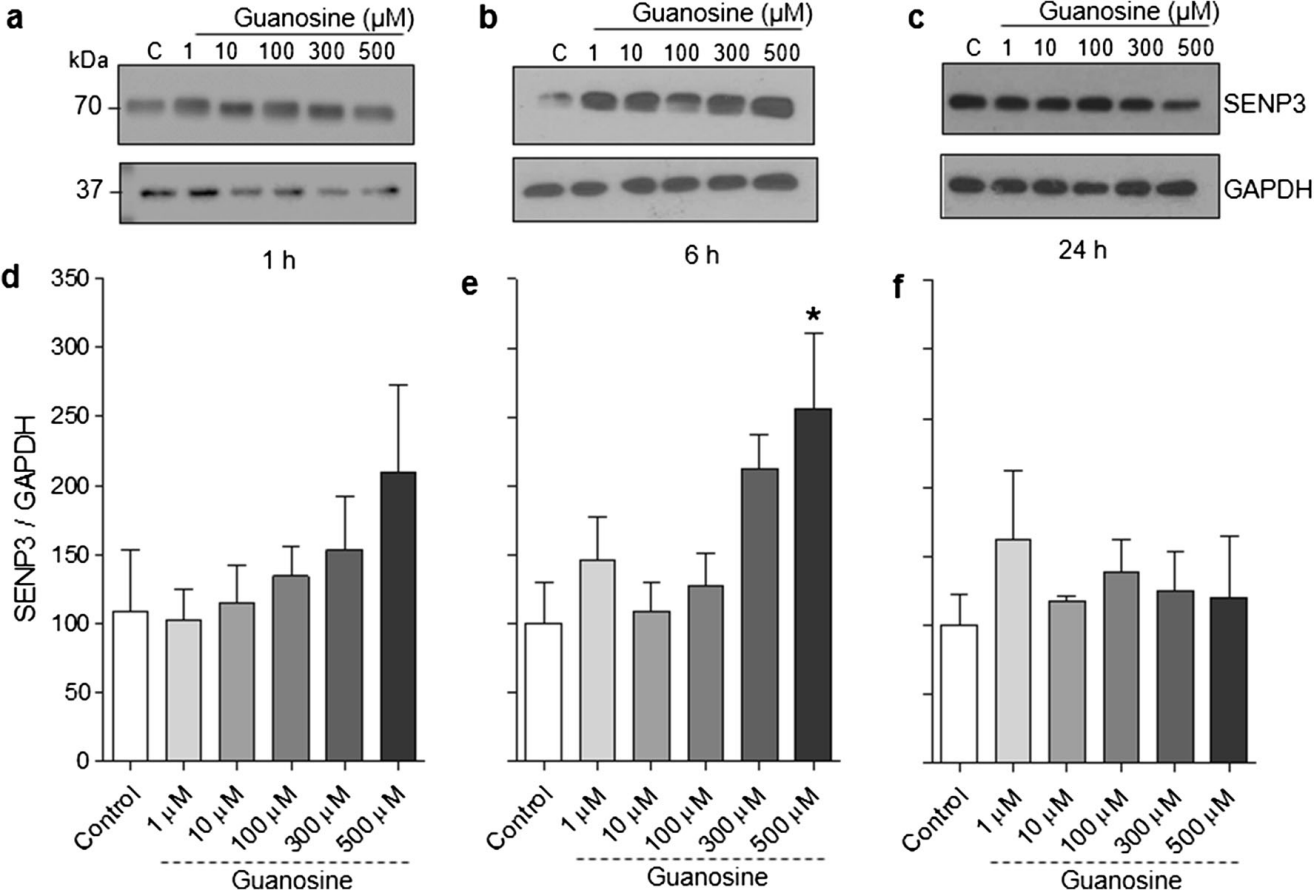
Guanosine increases neuronal SENP3 levels. Representative SENP3 Western blots from neurons treated with guanosine (1–500 μM) for **a** 1 h, **b** 6 h, and **c** 24 h. Optical density quantification of SENP3 for **d** 1 h, **e** 6 h, and **f** 24 h. GAPDH was used as a loading control. Results expressed as mean + standard error of the mean (*n* = 4 independent experiments). One-way ANOVA followed by Newman-Keuls multiple comparison analyses (*p < 0.05, vs. control). C, control; kDa, kilodaltons

Although SUMOylation is a PTM known to be involved in a wide range of cellular functions [2, 3, 48], very few modulators of SUMOylation have been identified. Here we aimed to evaluate the putative modulatory effect of guanosine on SUMOylation. Guanosine (10, 100, 300, or 500 μM) increased global SUMO2/3 conjugation in neurons at 1 h (Fig. 1 a and e, *F* = 5014; *p* = 0.001). However, increased global SUMO2/3 conjugation was not observed at longer time points (Fig. 1 b–d), suggesting the effect of guanosine is transient and subsides by 6 h. Moreover, we also observed an increase in levels of the SUMO protease SENP3, which removes SUMO2/3 from target proteins [49], after 6 h of treatment with 500 μM guanosine, suggesting the transient nature of the guanosine-induced increase in SUMO2/3 conjugation may in part be due to compensatory increases in SUMO protease expression (Fig. 2 b and e, *F* = 3638; *p* = 0.018).

**Fig. 3:**
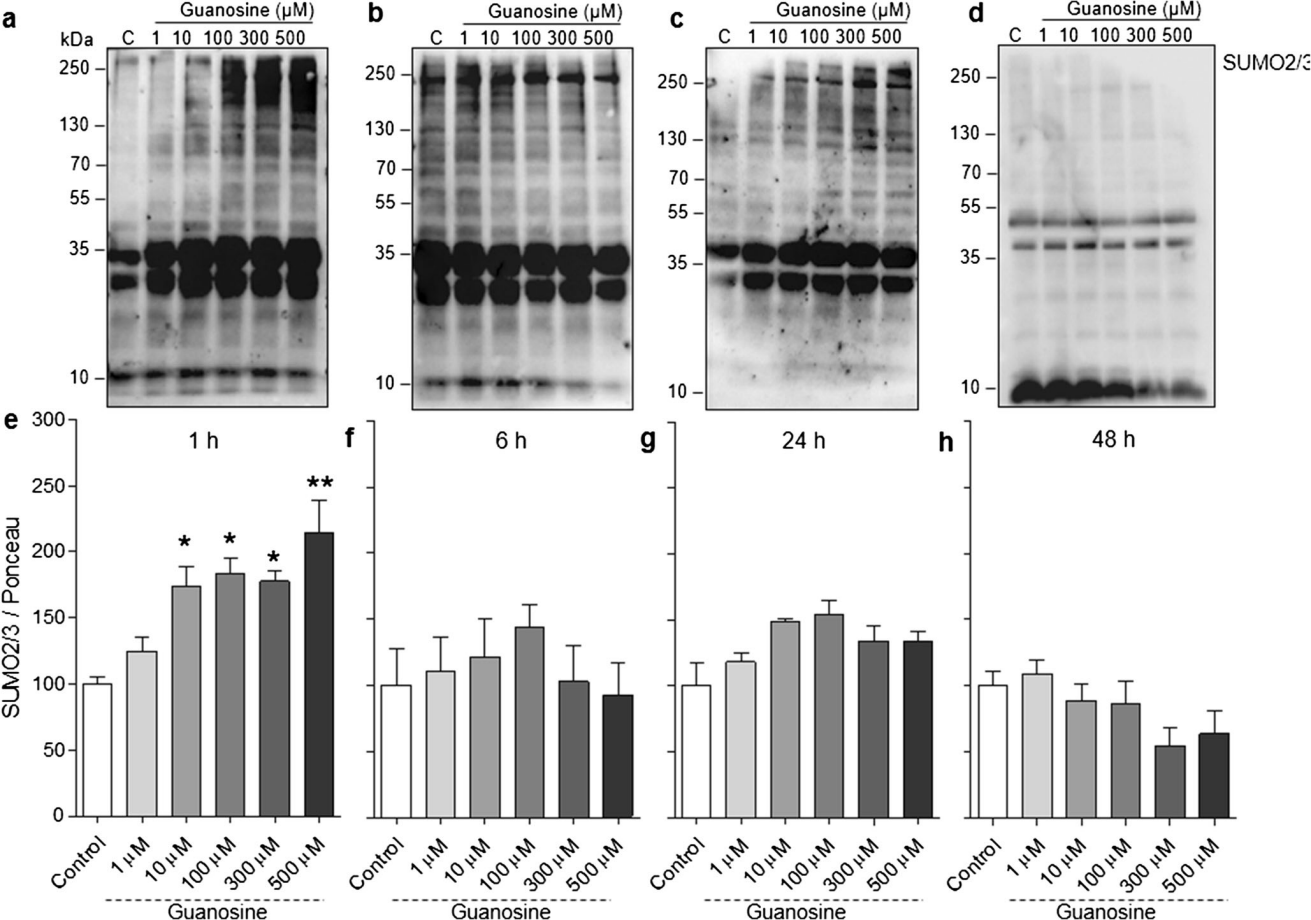
Guanosine increases global SUMO2/3 conjugation in astrocytes. Representative SUMO2/3 Western blots from astrocytes treated with guanosine (1–500 μM) for **a** 1 h, **b** 6 h, **c** 24 h, and **d** 48 h. Optical density quantification of global SUMO2/3 conjugation for **e** 1 h, **f** 6 h, **g** 24 h, and **h** 48 h. Ponceau staining was used as a loading control (Supplementary Fig. 1 D). Results expressed as mean + standard error of the mean (*n* = 3–5 independent experiments). One-way ANOVA followed by Newman-Keuls multiple comparison analyses (*p < 0.05, **p < 0.01 vs. control). C, control; kDa, Kilodaltons

**Fig. 4:**
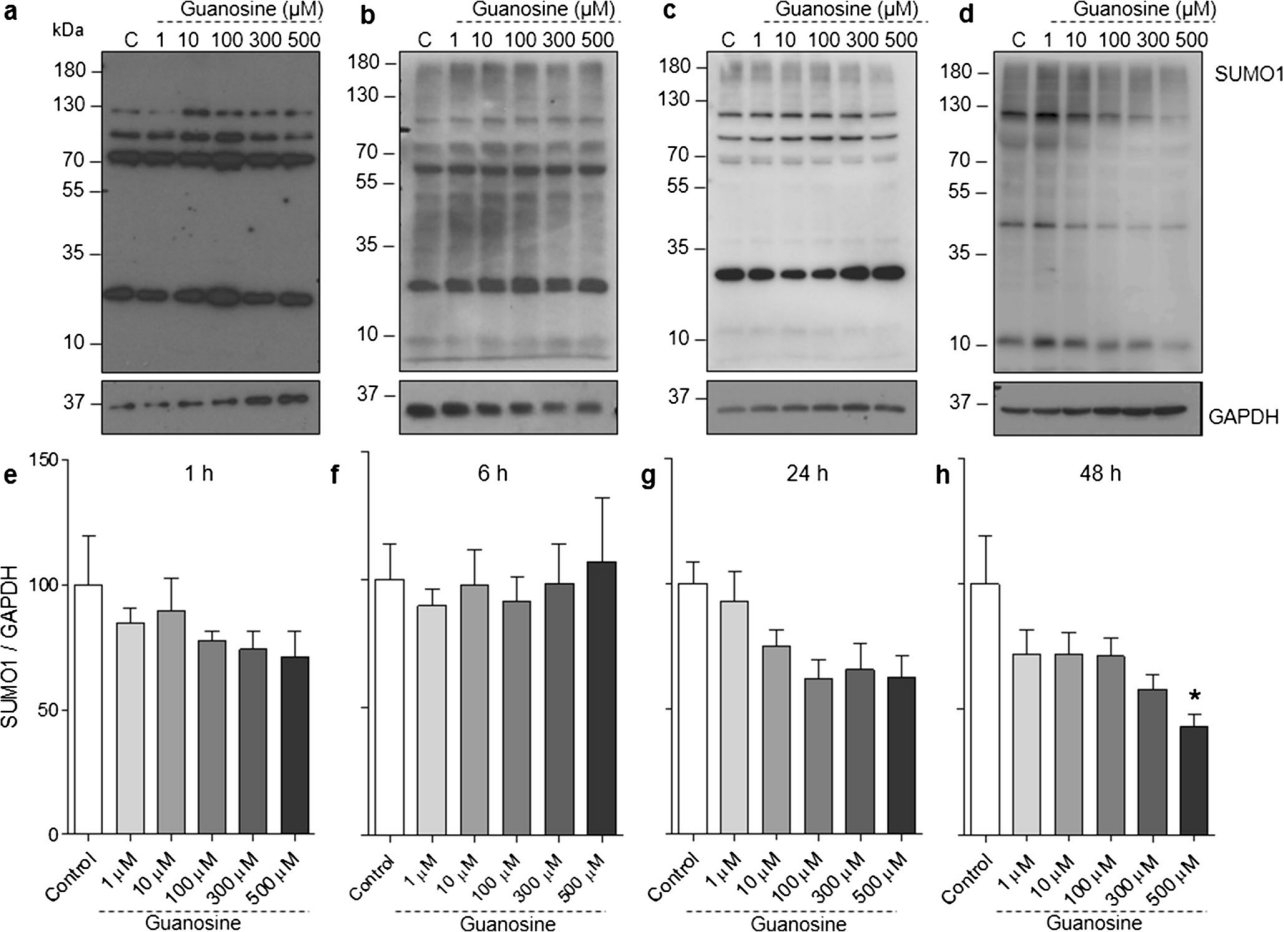
Evaluation of guanosine effects on global SUMO1 conjugation in neurons. Representative SUMO1 Western blots from neurons treated with guanosine (1–500 μM) for **a** 1 h, **b** 6 h, **c** 24 h, and **d** 48 h. Optical density quantification of global SUMO1 conjugation for **e** 1 h, **f** 6 h, **g** 24 h, and **h** 48 h. GAPDH was used as a loading control. Results expressed as mean + standard error of the mean (*n* = 3–5 independent experiments). One-way ANOVA followed by Newman-Keuls multiple comparison analyses (*p < 0.05 vs. control). C, control; kDa, kilodaltons

Considering the importance of astrocytes for the homeostasis and maintenance of neuronal function [50, 51], we also evaluated the effects of guanosine on protein SUMOylation in cortical astrocytes. Similarly to what we observed in neurons, guanosine increased global SUMO2/3 conjugation in astrocytes (at concentrations of 10, 100, 300, and 500 μM, 1 h) (Fig. 3 a and e, *F* = 9.025; *p* = 0.0002), but this effect was transient and not observed at later time points (Fig. 3 b–d). Together, these results demonstrate that guanosine can lead to a transient, reversible, increase in SUMO2/3 conjugation in both neurons and astrocytes. Conversely, however, guanosine (500 μM) decreased global SUMO1 conjugation in neurons at 48 h (Fig. 4 d and h, *F* = 2851; *p* = 0,047) and led to a similar trend towards decreased conjugation in astrocytes; however, this trend was not statistically significant (Supplementary Fig. 1 a–d).

### Guanosine does not affect cell viability of neurons or astrocytes

**Fig. 5:**
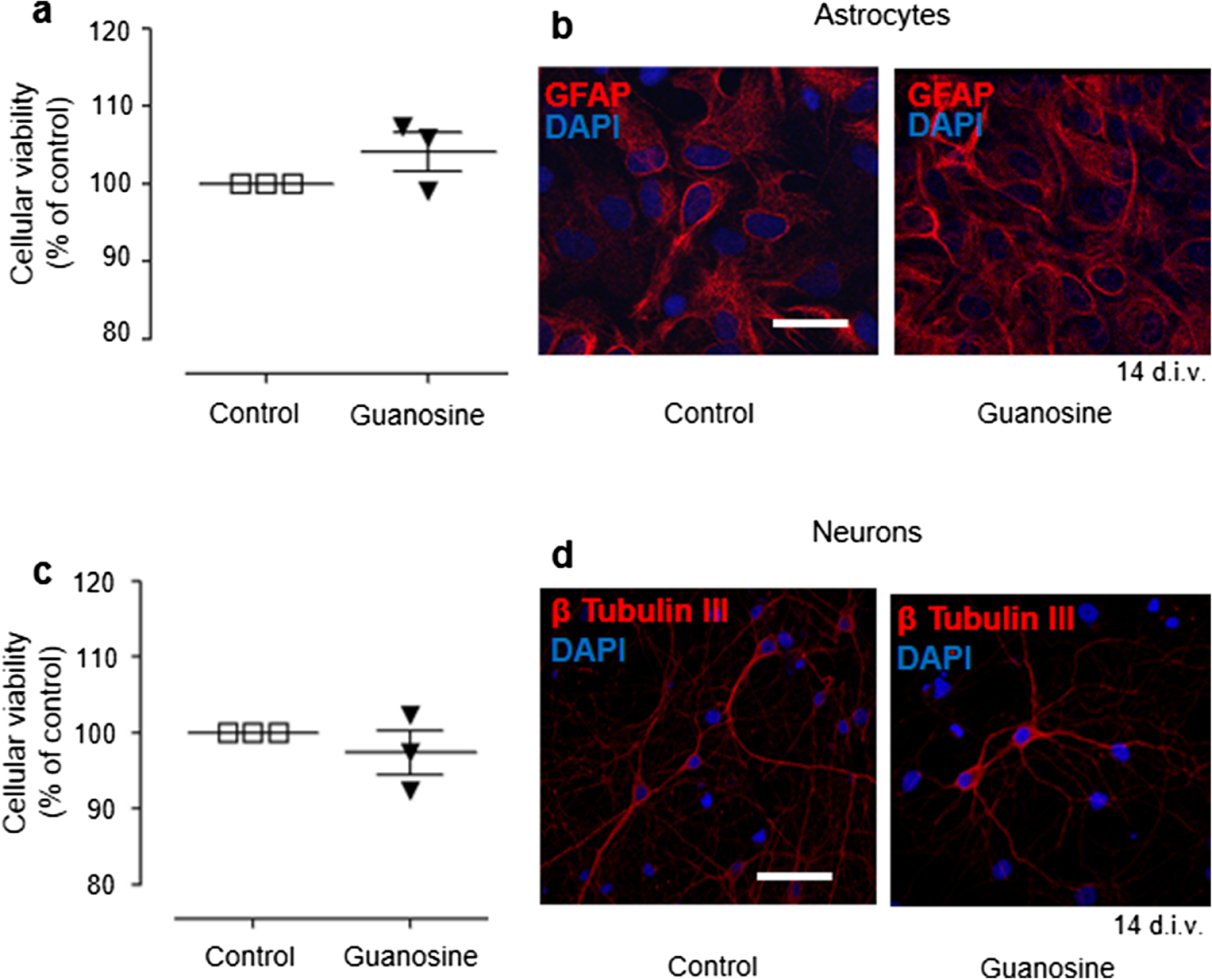
Guanosine does not affect astrocyte or neuronal viability and morphology. **a** Graph showing cellular viability (for MTT assay) of control and guanosine-treated astrocytes (500 μM, 1 h). **b** Confocal images of control and guanosine-treated astrocytes (100 μM, 48 h) stained for GFAP (glial fibrillary acidic protein, astrocytic marker) and DAPI (nuclear marker). **c** Graph showing cellular viability (for MTT assay) of control and guanosine-treated neurons (500 μM, 1 h). **d** Confocal images of control and guanosine-treated neurons (100 μM, 48 h) stained for β-tubulin III (neuronal marker) and DAPI (nuclear marker). Results expressed as mean ± standard error of the mean (*n* = 3 independent experiments). One-way ANOVA did not identify any significant differences

Since SUMOylation has been shown to be increased by a number of cellular stressors [52], we wanted to confirm that guanosine was not causing cellular stress and affecting cell viability. Importantly, 1 h treatment with 500 μM guanosine had no effect on cell viability in either astrocytes or neurons, as determined by MTT assay (Fig. 5 a and c, *F* = 2292; *p* = 0.1822). Furthermore, 48 h treatment with 100 μM guanosine did not obviously affect astrocyte or neuronal morphology, as determined by GFAP or β-tubulin III staining, respectively (Fig. 5 b and d). Together, these data demonstrate that guanosine can enhance global SUMO2/3 conjugation without adversely affecting cell health.

### Guanosine effects on SUMOylation are extracellular-mediated

**Fig. 6:**
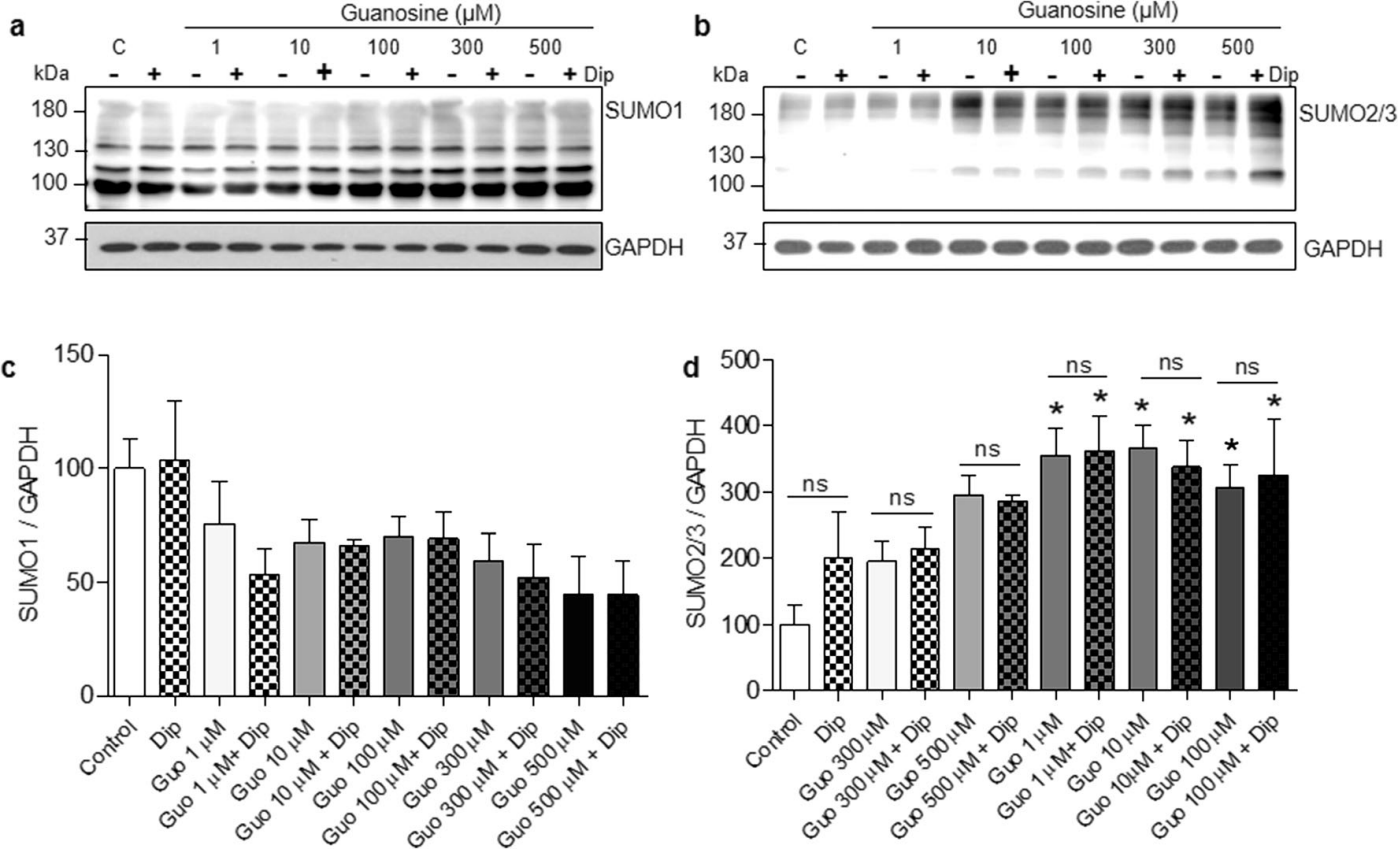
Guanosine-mediated increases in neuronal SUMO2/3 conjugation occur via extracellular mechanisms. **a** Representative Western blots of high molecular weight **a** SUMO1 and **b** SUMO2/3-conjugated proteins and their respective optical density quantifications in **c** and **d**. Neurons were co-incubated with dipyridamole (Dip, 10 μM) and guanosine (1, 10, 100, 300, and 500 μM) for 1 h. GAPDH was used as a loading control. Results expressed as mean + standard error of the mean (*n* = 3 independent experiments). One-way ANOVA followed by Newman-Keuls multiple comparison analyses indicates the effects of the treatments with guanosine. C, control; kDa, kilodaltons

Guanosine uptake is mediated through nucleoside transporters present in the cell membrane [53, 54]. In contrast, the SUMOylation proteins are intracellular components [3]. In order to investigate whether the mechanism by which guanosine increases global SUMOylation requires guanosine uptake, or is mediated by extracellular actions of guanosine, neurons were pre-incubated with dipyridamole (10 μM), a pan-inhibitor of nucleoside transporters. Twenty minutes later, guanosine (500 μM) was added in the presence of dipyridamole, and neurons were further incubated for an hour. As previously, SUMO1 conjugation remained unchanged (Fig. 6 a and c), but an increase in high molecular weight SUMO2/3 conjugates was observed both in the presence and absence of dipyridamole (Fig. 6 b and d, *F* = 3.444; *p* = 0.0055). These results suggest that the guanosine-induced increase in SUMO2/3 conjugation does not require guanosine internalization and indicates that guanosine is acting through a membrane receptor interaction.

### The effects of guanosine on protein SUMOylation are mediated by adenosine A1 receptors

**Fig. 7:**
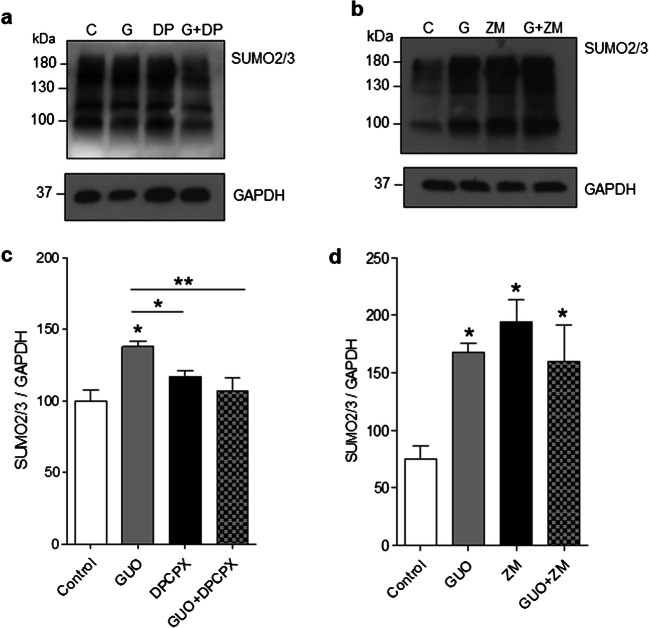
Guanosine increases neuronal SUMO2/3 conjugation via modulation of adenosine receptors. Neurons were co-incubated with guanosine (500 μM) and either **a** the adenosine A1 receptor antagonist DPCPX (100 nM) or **b** the A2A receptor antagonist ZM241385 (ZM) for 1 h, followed by Western blotting for SUMO2/3. Quantification of data is shown in **c** (for DPCPX) and **d** (for ZM241385). One-way ANOVA followed by Newman-Keuls multiple comparison analyses indicates the effects of the treatments with guanosine (*p < 0.05 vs. control). C, control; G, guanosine; DP, DPCPX; kDa, kilodaltons

Next, we sought to investigate whether the guanosine-induced increase in global SUMO2/3 conjugation was mediated by A1 and/or A2A adenosine receptors. Neurons were treated with guanosine (500 μM) in the presence or absence of the A1 receptor antagonist DPCPX (100 nM) for 1 h. DPCPX abolished the effect of guanosine on SUMO2/3 conjugation (Fig. 7 a and c, *F* = 3.086; *p* = 0.0571), suggesting guanosine is enhancing SUMO2/3 conjugation through A1 receptors. In the same way, neurons were treated with guanosine in the presence or absence of the A2A antagonist ZM241385 (50 nM) for 1 h. ZM241385 did not affect the guanosine-induced increase in SUMO2/3 conjugation (Fig. 7 b and d, *F* = 6.282; *p* = 0.0051). However, ZM241385 per se increased global SUMO2/3 conjugation (Fig. 7 b and d). Surprisingly, adenosine treatment, at the same concentrations used for guanosine (1, 10, 100, 300, and 500 μM), did not affect global protein SUMOylation by either SUMO1 (Supplementary Fig. 2 a and c, *F* = 0.371; *p* = 0.85) or SUMO2/3 (Supplementary Fig. 2 b and d, *F* = 2.220; *p* = 0.08).

## Discussion

Protein SUMOylation is a highly dynamic PTM [3, 55, 56]. Here, we demonstrate that guanosine induces an increase in global protein SUMO2/3 conjugation after 1 h stimulation with concentrations of guanosine of 10 μM or higher. This effect was not observed at longer time points, suggesting this effect is transient and may ultimately be counteracted by a concomitant increase in levels of SENP3, a deSUMOylating enzyme that shows preference for deconjugating SUMO2/3 over other SUMO isoforms [49]. We cannot rule out the possibility that SUMO2/3 conjugation could return to control levels earlier than 6 h; however, this will require further investigation. The consequences of both neuronal and astrocytic increases in SUMO2/3 conjugation after 1 h guanosine treatment, and the decrease in neuronal SUMO1 conjugation at 48 h, need to be further investigated. However, importantly, we have demonstrated that guanosine stimulation does not lead to an observable loss of cellular viability or alteration of cell morphology.

A growing number of studies have demonstrated the importance of SUMO2/3 conjugation in mediating neuroprotective mechanisms [8, 57–59]. In a recent screen for compounds with SENP2 inhibitory activity, 6-thioguanine, which increases SUMO1-ylation and SUMO2/3-ylation levels, and the compound isoprenaline, which increases SUMO2/3-ylation levels, were found to protect SH-SY5Y cells from oxygen and glucose deprivation, an in vitro model of ischemia [60]. As both 6-thioguanine [60] and guanosine, which was used in our study, increased SUMO2/3 conjugation, this effect may be related to similarities in their chemical structures. However, since our data demonstrate that guanosine uptake is not required for its effects on protein SUMOylation, it seems unlikely that the effects we observe are due to inhibition of intracellular SENP2.

Previous evidence has shown that there is more SUMO2/3 available to be conjugated to target proteins than SUMO1, which may explain the selective increase in SUMO2/3 conjugation at 1 h [52]. In Cos7 cells, there is approximately 40% more free SUMO2/3 than SUMO1 [52, 61, 62]; however, whether this is the case in neurons and astrocytes is not known. It is important to point out that some substrates may be modified only by SUMO1, or SUMO2/3, or both [63]. In this context, future investigations focusing on which cellular targets are being SUMOylated upon guanosine treatment will provide important information as to the functional consequences of the guanosine-induced increase in SUMO conjugation.

Under physiological conditions, extracellular guanosine is efficiently internalized [64]. Since the SUMOylation machinery is present intracellularly [3], we evaluated whether the effects of guanosine on SUMO conjugation required its internalization. Using dipyridamole to block guanosine internalization, we still observed an increase in SUMO2/3 conjugation in neurons. Similarly, in previous studies, blockade of nucleoside transporters did not impair guanosine-mediated prevention of apoptosis in cultured rat astrocytes [32], or the neurotrophic effects of guanosine in primary cultured cerebellar neurons [65]. These results strongly suggest that guanosine exerts its protective effects via an extracellular mechanism, which likely involves membrane receptor activation.

Previous studies from our group have demonstrated extracellular effects of guanosine acting through A1 and A2A adenosine receptors [30]. In the present study, A1 receptor blockade by DPCPX abolished the guanosine-induced increase in SUMO2/3 conjugation, suggesting that guanosine might be acting via A1 receptors. Regarding A2A receptor modulation with ZM241385, the antagonist itself increased SUMO2/3 conjugation, in a similar manner to guanosine, and co-incubation of ZM241385 plus guanosine did not further increase SUMO2/3-ylation compared with either compound alone. The effect of ZM241385 in enhancing SUMOylation per se suggests that constitutive A2A receptor activity might be directly modulating endogenous SUMO2/3 conjugation; however, this needs to be further experimentally confirmed. ZM241385 is classically described as an A2A receptor antagonist; however, some studies suggest that it can act also as an inverse agonist for A2A receptors [36, 66–68], which could explain its ability in promoting such an effect. Regarding the effect of guanosine, it is feasible that it may also act by reducing A2A receptor activity, similar to ZM241385, since we have previously shown its neuroprotective effect is not observed in A2A receptor-knockout mice [69]. Additionally, binding and functional studies in HEK293 cells transfected with A1 and A2A receptors showed guanosine did not interfere with A1 receptor-mediated signaling, and that it modulated A2A receptor binding and intracellular signaling only in cells co-expressing A1 and A2A receptors, providing the first piece of evidence that the effects of guanosine may occur through interaction with an oligomeric organization of adenosine receptors, namely the A1R-A2AR heteromer [69]. However, the exact mechanism of guanosine action is still unknown. Indeed, since guanosine reportedly shows low affinity for adenosine receptors [24], it remains possible that guanosine acts via other receptor proteins [70], or via promoting the release of endogenous adenosine, as has been reported in some non-neural cell types [71, 72]. Nonetheless, our data support a model whereby adenosine receptor activity is required for the effects of guanosine in promoting SUMOylation in neurons.

Since guanosine increased SUMO2/3 conjugation via adenosine receptors, we hypothesized that adenosine would promote a similar effect. Surprisingly, adenosine (1, 10, 100, 300, and 500 μM) for 1 h did not modulate global SUMO2/3 or SUMO1 conjugation. However, it remains possible that adenosine could modulate SUMOylation at shorter time points, especially considering that G protein-coupled receptors, such as adenosine receptors, may suffer from desensitization and internalization in response to continuous exposure to agonist, preventing the observation of downstream effects [73–75]. Another possible explanation is that guanosine may not promote desensitization of adenosine receptors since it is not their endogenous agonist. In addition, it has been suggested that guanosine can act as an allosteric modulator at adenosine receptors [24], suggesting it may therefore produce different effects to direct agonist activation with adenosine; however, this still needs further experimental confirmation.

To the best of our knowledge, there is, to date, only one experimental demonstration that SUMOylation can be modulated through adenosinergic signaling. The protein IκBα (nuclear factor of kappa light polypeptide gene enhancer in B-cells inhibitor, alpha), an important modulator of inflammatory responses, can be SUMO1-ylated in response to adenosine signaling. Following hypoxia and reoxygenation, there was an increase in SUMO1 conjugation to IκBα in HeLa cells, and treatment with NECA, a non-specific adenosine receptor agonist, increased SUMO1 conjugation to IκBα in a concentration-dependent manner. In contrast, a nonselective adenosine receptor antagonist, 8-phenyltheophylline, abolished NECA-induced IκBα SUMO1-ylation [76]. However, exactly how adenosine receptor activation leads to enhanced SUMOylation of IκBα is unknown.

## Conclusion

The importance of the SUMOylation pathway for neuronal function and dysfunction is well demonstrated in the literature [3, 77, 78]. Here we show for the first time that guanosine can increase global SUMO2/3 conjugation in neurons, in a manner that does not require its uptake into cells and which is dependent on the modulation of adenosine receptors, most likely the A1-A2A receptors. Despite much interest in SUMO as a possible therapeutic target, non-toxic modulators of SUMOylation are still scarce, although a few chemical compounds such as TAK-981 [79], ginkgolic acid [80], and tannic acid [81] have been shown to exert effects on global SUMO conjugation. Taken together, our results suggest that guanosine, an endogenous neuromodulator [23, 24, 82, 83], can lead to enhanced SUMO2/3-ylation, a phenomenon that has been shown to be neuroprotective against a number of cell stressors [3, 9, 13, 14, 16, 84]. Our findings therefore highlight guanosine as a potential therapeutic strategy to promote neuronal and glial survival.

## Electronic supplementary material

ESM 1 Evaluation of guanosine effects on global SUMO1 conjugation in astrocytes. Representative SUMO1 Western blots from astrocytes treated with guanosine (1 – 500 μM) for **a.** 1 h, **b.** 6 h, **c.** 24 h and **d.** 48 h. SUMO1 conjugation optical density quantification for **e.** 1 h, **f.** 6 h, **g.** 24 h and **h.** 48 h. (PNG 1130 kb)

High Resolution Image (TIF 282 kb)

ESM 2 Ponceau staining was used as a loading control. Representative Ponceau staining used to normalize the Western blots for global SUMO1-conjugated proteins in astrocytes at **i.** 1 h, **j.** 6 h, **k.** 24 h and **l.** 48 h. Representative Ponceau staining used to normalize the Western blots for global SUMO2/3-conjugated proteins in astrocytes at **m.** 1 h, **n.** 6 h, **o.** 24 h and **p.** 48 h. Results expressed as mean + standard error of the mean (*n* = 3-5 independent experiments). One-way ANOVA did not identify any significant differences between groups. C: Control. kDa: Kilodaltons. (PNG 1493 kb)

High Resolution Image (TIF 409 kb)

ESM 3 Adenosine does not change global SUMO1 nor SUMO2/3 conjugation in neurons at 1 h. Representative Western blots of **a.** SUMO1 and **b.** SUMO2/3 conjugation and optical density quantifications for **c.** SUMO1 and **d.** SUMO2/3 from neurons treated with adenosine (1 – 500 μM) for 1 h. GAPDH was used as a loading control. Results expressed as mean + standard error of the mean (n= 3-5 independent experiments). One-way ANOVA did not identify any significant differences between groups. C: Control. kDa: Kilodaltons. (PNG 574 kb)

High Resolution Image (TIF 122 kb)
