# Supplementary figures and images for: Guanosine modulates SUMO2/3-ylation in neurons and astrocytes via adenosine receptors
Source: Purinergic Signal. 2020 Sep 5;16(3):439–50. doi: 10.1007/s11302-020-09723-0 (PMC7524998; doi:10.1007/s11302-020-09723-0)

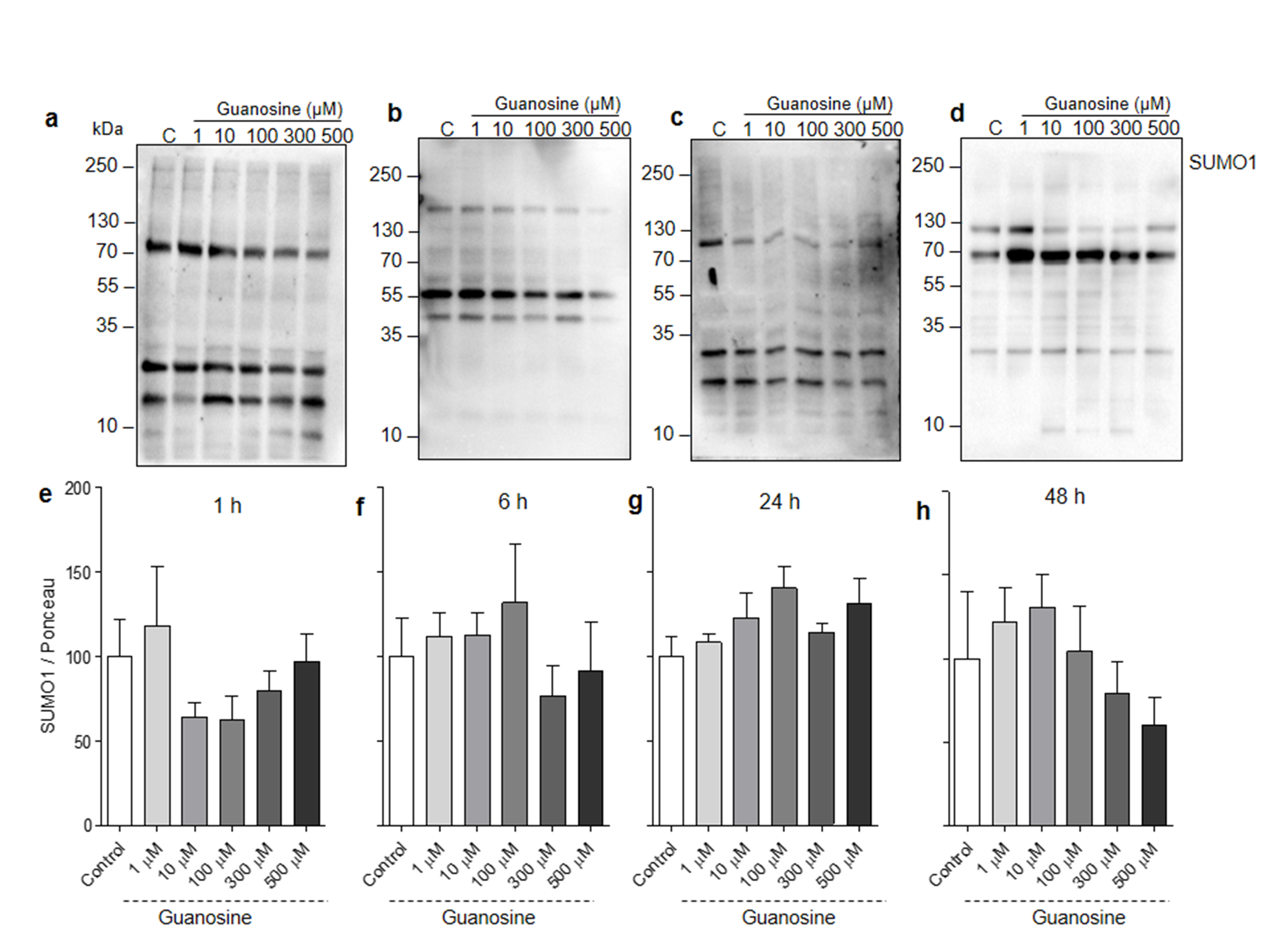

Supplement: Supplementary file 1 — Evaluation of guanosine effects on global SUMO1 conjugation in astrocytes. Representative SUMO1 Western blots from astrocytes treated with guanosine (1 – 500 μM) for a. 1 h, b. 6 h, c. 24 h and d. 48 h. SUMO1 conjugation optical density quantification for e. 1 h, f. 6 h, g. 24 h and h. 48 h. (PNG 1130 kb) [file 11302_2020_9723_Fig8_ESM.png]

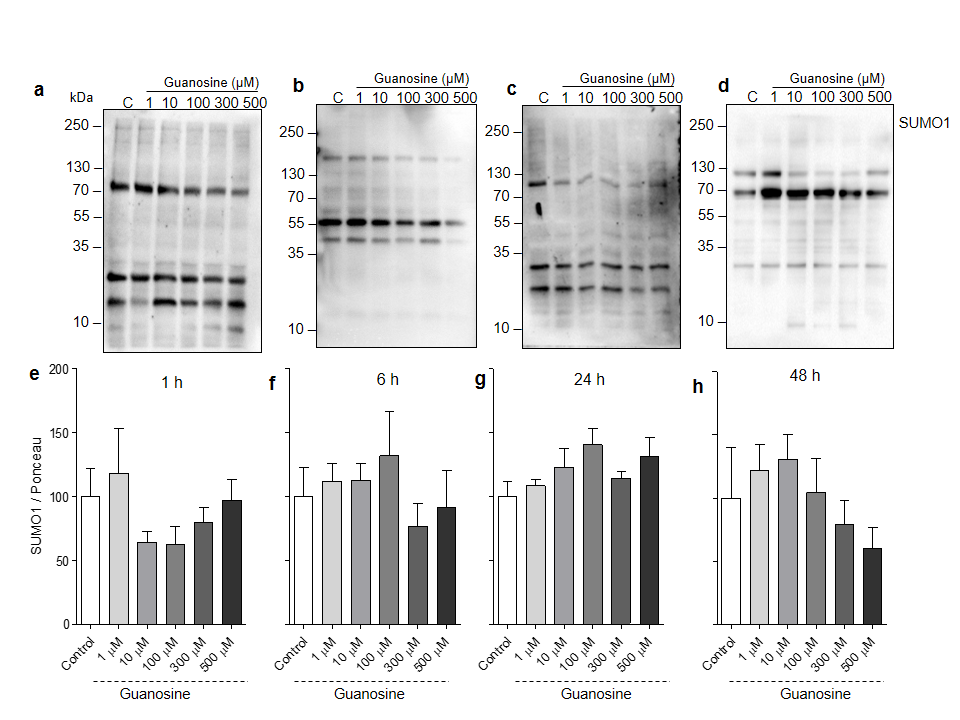

Supplement: Supplementary file 2 — High Resolution Image (TIF 282 kb) [file 11302_2020_9723_MOESM1_ESM.tif]

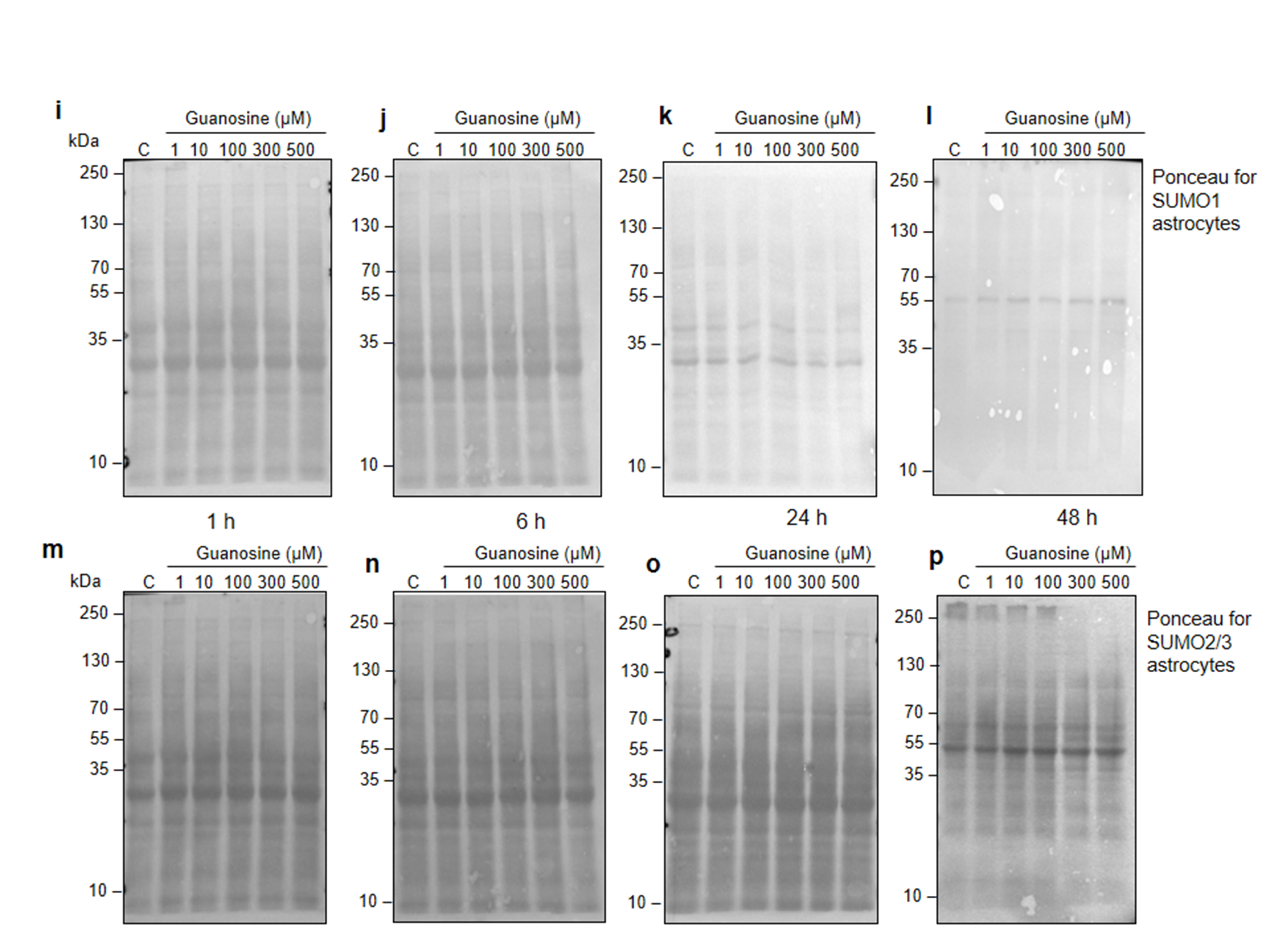

Supplement: Supplementary file 3 — Ponceau staining was used as a loading control. Representative Ponceau staining used to normalize the Western blots for global SUMO1-conjugated proteins in astrocytes at i. 1 h, j. 6 h, k. 24 h and l. 48 h. Representative Ponceau staining used to normalize the Western blots for global SUMO2/3-conjugated proteins in astrocytes at m. 1 h, n. 6 h, o. 24 h and p. 48 h. Results expressed as mean + standard error of the mean (n = 3-5 independent experiments). One-way ANOVA did not identify any significant differences between groups. C: Control. kDa: Kilodaltons. (PNG 1493 kb) [file 11302_2020_9723_Fig9_ESM.png]

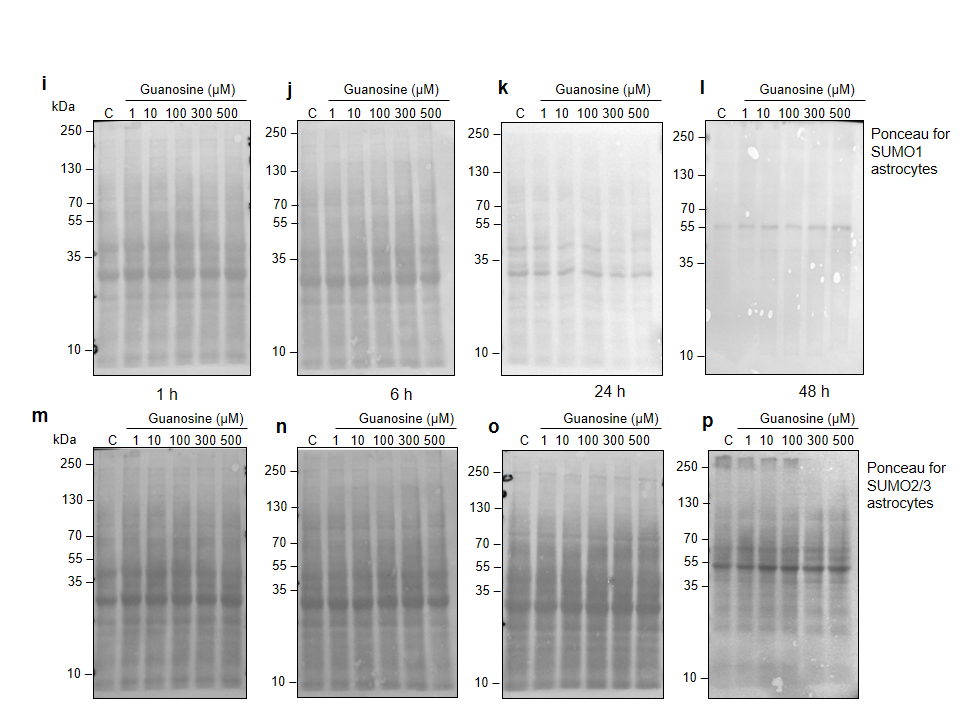

Supplement: Supplementary file 4 — High Resolution Image (TIF 409 kb) [file 11302_2020_9723_MOESM2_ESM.tif]

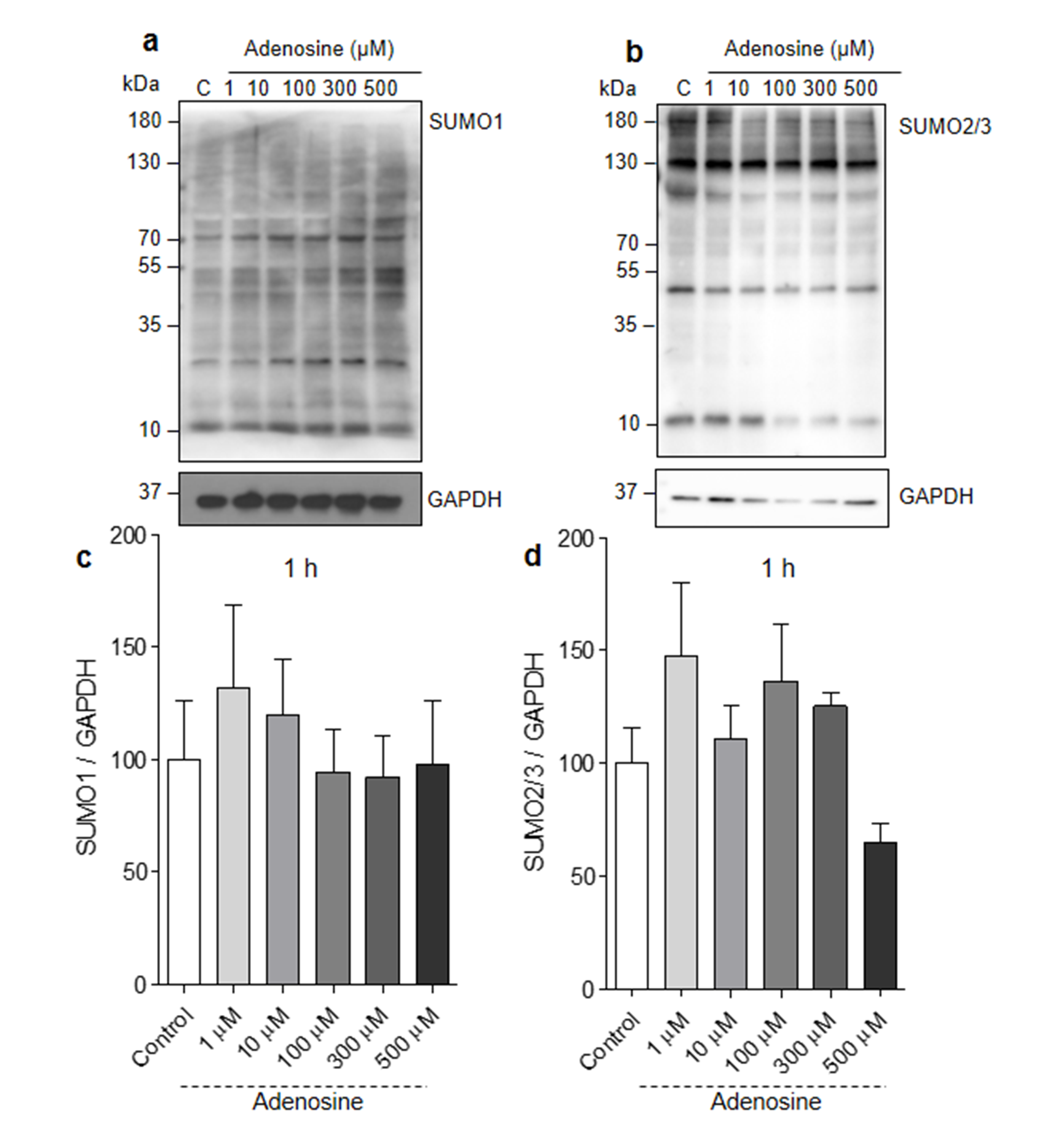

Supplement: Supplementary file 5 — Adenosine does not change global SUMO1 nor SUMO2/3 conjugation in neurons at 1 h. Representative Western blots of a. SUMO1 and b. SUMO2/3 conjugation and optical density quantifications for c. SUMO1 and d. SUMO2/3 from neurons treated with adenosine (1 – 500 μM) for 1 h. GAPDH was used as a loading control. Results expressed as mean + standard error of the mean (n= 3-5 independent experiments). One-way ANOVA did not identify any significant differences between groups. C: Control. kDa: Kilodaltons. (PNG 574 kb) [file 11302_2020_9723_Fig10_ESM.png]

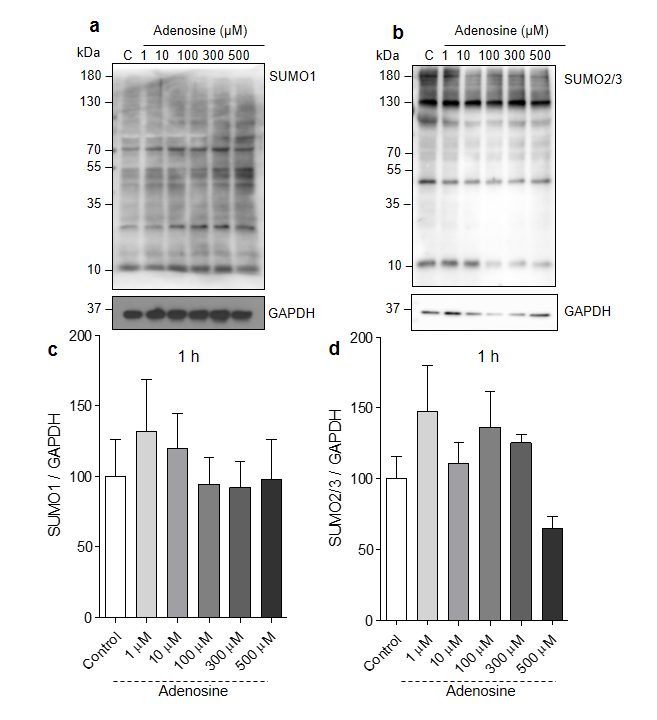

Supplement: Supplementary file 6 — High Resolution Image (TIF 122 kb) [file 11302_2020_9723_MOESM3_ESM.tif]
